# The Function of *Ophiocordyceps sinensis* in Airway Epithelial Cell Senescence in a Rat COPD Model

**DOI:** 10.1155/2018/6080348

**Published:** 2018-04-01

**Authors:** Xiaohui Ma, Xingai Jiao, Jinxiang Wu, Jiping Zhao, Yurong Xu, Tian Liu, Jiawei Xu, Lei Yang, Liang Dong

**Affiliations:** ^1^Department of Respiratory Medicine, Qilu Hospital of Shandong University, Jinan, Shandong, China; ^2^Department of Respiratory Medicine, Shandong Chest Hospital, Jinan, Shandong, China

## Abstract

*Ophiocordyceps sinensis* (*O. sinensis*) seems to be able to alleviate airway epithelial cell senescence in chronic obstructive pulmonary disease (COPD). The objective of the study is to evaluate the effect of *O. sinensis* on airway epithelial senescence in the COPD model both in vitro and in vivo. We observed the expression of P16 and P21 in the airway epithelia of 30 patients with COPD. The optimal concentration of *O. sinensis* and exposure time of the cigarette smoke extract (CSE) were determined in vitro, and senescence-associated *β*-galactosidase (SA-*β*-gal) and 5-bromodeoxyuridine (BrdU) were used to evaluate the senescence and proliferation of human bronchial epithelial (16HBE) cells pretreated with *O. sinensis* by staining kits. COPD model rats were treated with *O. sinensis* at various concentrations to determine the changes in P16 and P21 expression in airway epithelial tissues. It was found that the expression levels of P16 and P21 were higher in the airway epithelia of COPD patients than those in the control group based on immunohistochemical staining, real-time quantitative PCR, and western blotting. The CSE could induce 16HBE cell senescence, and *O. sinensis* could alleviate CSE-induced senescence and promote the proliferation of 16HBE cells. The expression levels of P16 and P21 were also higher in the airway epithelia of COPD model rats; however, the levels of P16 and P21 in the groups treated with all concentrations of *O. sinensis* were obviously lower than those in the COPD model group based on real-time quantitative PCR and western blotting. In conclusion, the CSE can induce airway epithelium senescence, and *O. sinensis* can inhibit CSE-induced cellular senescence, both in vitro and in vivo.

## 1. Introduction

Chronic obstructive pulmonary disease (COPD) is characterized by an expiratory airflow limitation that is not fully reversible and is associated with an anomalous inflammatory reaction, mainly in response to tobacco smoke. Chronic inflammation is a major histological characteristic of COPD patients [1]. At the same time, senescence plays an important role in the pathogenesis of chronic lung diseases including COPD and lung fibrosis as well [2]. Previous reports have demonstrated the critical role of cellular senescence in respiratory diseases, such as asthma, pulmonary fibrosis, and lung cancer, especially in COPD [3–5].

Cellular senescence is a fundamental property of all cells in which cell growth is arrested under conditions of cellular stress [6]. In general, cellular senescence can be viewed as a protective mechanism against cancer. However, aging and cellular senescence underlie chronic diseases, involving, for instance, the brain (e.g., Alzheimer's disease), the cardiovascular system (e.g., atherosclerosis), and the musculoskeletal system (e.g., osteoporosis), and COPD [4]. Cellular senescence is associated with decreased physiological function and protein degradation and is irreversible [7]. In particular, cellular senescence affects regeneration ability by reducing the amount of hematopoietic stem cells, and aging cells release cytokines to further enhance the inflammatory response, which leads to airway inflammation and airway remodeling in COPD patients [8, 9]. It is not surprising that cellular senescence is induced in the airway epithelial cells of COPD patients. However, the mechanism of cell senescence in COPD is not clear. Therefore, we demonstrated that cellular senescence is required in COPD.

*Ophiocordyceps sinensis* (*O. sinensis*) has been used in traditional Chinese medicine for a long history. Many bioactive constituents, such as polysaccharides, cordycepin, mannitol, aminophenol, and ergosterol, have been found in *O. sinensis* [10]. Chemical constituents extracted from *O. sinensis* have various pharmacological actions, including nephroprotective, hepatoprotective, anti-inflammatory, antioxidative, and antiapoptotic effects [10]. In COPD patients, artificially cultivated *O. sinensis* can inhibit airway inflammation, improve lung function, correct airway Th1/Th2 ratio imbalance and two-way immune regulation, alleviate respiratory muscle fatigue, and increase exercise tolerance [11]. However, the effects of *O. sinensis* on cell senescence in COPD have not been reported although its functional roles have been widely documented. Based on these previous studies, we hypothesize that the cigarette smoke extract (CSE) can induce airway epithelial senescence in COPD, and *O. sinensis* can alleviate CSE-induced cellular senescence. Therefore, this study investigates how *O. sinensis* regulates cellular senescence in COPD airway epithelium in vivo and in vitro.

## 2. Materials and Methods

### 2.1. Ethics Statement

The research was approved by the ethics review committee for human studies at Qilu Hospital, Shandong University, Jinan City, China. All biopsy specimens were obtained from the participants after informed consent was obtained.

### 2.2. Patients

All biopsy tissues were obtained from the Qilu Hospital of Shandong University (Jinan, Shandong, China) between September 2013 and December 2015. Thirty COPD specimens were obtained from lung cancer resections, and nontumor lung tissues were collected when patients were diagnosed with COPD. Thirty normal lung tissues were obtained from body donations because of trauma or death; these individuals had no obvious pulmonary disease, and the samples were used as a control group.

As shown in Table 1, the age and gender of patients in the COPD group and the control group were similar. Pulmonary function tests were performed based on the predicted forced expiratory volume (FEV1%), the predicted forced vital capacity (FVC%), and the ratio FEV1/FVC (%). The predicted FEV1% and the FEV1/FVC (%) were significantly lower in patients with COPD than those in non-COPD subjects (*p* < 0.05).

### 2.3. Cells and Reagents

*O. sinensis* liquid was obtained from Hangzhou Sino-US East China Pharmaceutical Co., Ltd (Hangzhou, China). Artificially cultured *O. sinensis* powder was dissolved in normal saline (500 mg/ml) to prepare the turbid liquid suspension. Septwolves cigarettes (tobacco type, tar: 14 mg, nicotine content: 1.2 mg, and carbon monoxide fumes: 15 mg) were obtained from Longyan Tobacco Industry LLC (Longyan, China). Lipopolysaccharides (LPS, L3129) were purchased from Sigma Company (Los Angeles, USA). The 16HBE (human bronchial epithelioid) cell line was purchased from CHI Scientific, Inc. (Shanghai, China).

The 16HBE cells were cultured for six days in high-glucose Dulbecco's modified Eagle's medium (H-DMEM) in 48-well plates (Invitrogen, Life Technologies, New Zealand) containing 10% Gibco® fetal bovine serum (FBS, Invitrogen) at 37°C in a 5% CO_2_ atmosphere. Each day, 100 *μ*l of the medium per well was removed and was replaced with fresh medium. The preparation of the CSE was previously described [3, 5]. When cell density was 75%, a CSE solution was added to the 48-well plates at various concentrations (0, 0.5%, 1.0%, 2.0%, and 5.0%; 0.05 ml), and the cells were cultured for an additional 24 hours. Total proteins were extracted for western blotting to the optimal CSE solution concentration screening. When the optimal concentration of the CSE solution (0.05 ml) was added to the cells for durations of 0, 3, 6, and 12 hours (Supplementary Figure 1), the total extracted protein was used to detect P16 and P21 expression by western blotting to determine the best reaction time. To determine the effect of *O. sinensis* on cells, 16HBE cells were pretreated with *O. sinensis* (100 *μ*g/ml) [5] for 2 hours, and the cells were then cultured with the optimal concentration of the CSE solution for 6 hours. The cells of different treatment groups (control group, CSE group, and CSE + *O. sinensis* group) were collected. In the preliminary experiment, we have found that *O. sinensis* (100 *μ*g/ml) does not affect the gross cell morphology, proliferation rate, and apoptosis rate.

### 2.4. Animals and COPD Models

Male Wistar rats (200 ± 20 g) were purchased from the Shandong University Experimental Animal Center. The animals were housed in a temperature of −37°C and humidity-controlled conditions and kept on a 12 h/12 h light/dark cycle, with free access to regular chow and water. The rats were randomly divided into five groups with fifteen rats per group, as described below: control group (CON), COPD group, COPD + low dose of the *O. sinensis* group (LOW, 2.5 g/kg/day), COPD + intermediate dose of the *O. sinensis* group (MID, 5.0 g/kg/day), and COPD + high dose of the *O. sinensis* group (HIG, 7.5 g/kg/day).

The experimental protocol was approved by the Experimental Animal Care and Ethics Committee at the Qilu Hospital of Shandong University, Jinan, China. The detailed procedures for the COPD model rats were described in a previous report [12, 13]. Briefly, the rats were anesthetized with intraperitoneal ketamine (35 mg/kg) and were administered LPS twice using intratracheal instillation on day 1 (0.2 ml, 1 mg/ml) and day 14 (0.1 ml, 1 mg/ml). The rats were exposed to tobacco smoke from 8 cigarettes for each treatment twice per day during the first two weeks and were then exposed to fifteen cigarettes per treatment three times per day from week 3 to week 12. The rats were placed in a special sealed box connected to a smoke source to receive two or three 30-minute exposures per day at an interval of one to two hours. The control rats were exposed to the air following similar procedures. Different doses of the *O. sinensis* liquid were given by intragastric administration for 14 days to the COPD rats three times a day after 12 weeks of exposure to tobacco smoke. The control group and COPD group rats were administrated by the same amount of normal saline.

### 2.5. Histological Analysis

The lung tissue blocks from COPD patients, control humans, and each rat group were immersed in 4% paraformaldehyde, embedded in paraffin, and cut into 5 *μ*m thick sections. Lung tissue paraffin sections were prepared for HE staining and immunohistochemical assays. The following primary antibodies were used: anti-p16 (f-12, SC-1661), anti-p21 (C-19, SC397), and *β*-actin (C4, SC47778). All antibodies were purchased from Santa Cruz Biotechnology, Inc. (Santa Cruz, CA, USA). The dilution rate for both of the primary antibodies used in the immunohistochemical assay was 1 : 150. We count the optical density (OD) of every group's immunohistochemical sections with the software Image-Pro Plus. Briefly, all the pictures were equalized by the “the best equalization” function of the software firstly, and then, the OD values were counted. The details can be seen in the operation manual for the software.

### 2.6. Western Blotting Analysis

Lung tissues from every group and the human airway epithelial cell line (16HBE) were preserved at −80°C and thawed on ice before use. Total proteins in lung tissue samples and 16HBE cells were extracted prior to western blotting. P16 and P21 antibodies were prepared for the western blotting analysis. The dilution rate for both of the primary antibodies was 1 : 1000.

### 2.7. Real-Time Fluorescence Quantitative PCR

RNAiso plus, RT reagent kit, Premix Ex Taq Version 2.0, and SYBR Green real-time PCR master mix were purchased from Takara Company (Dalian, China) and were used to perform the real-time quantitative PCR assay. Briefly, total RNA was extracted with RNAiso plus, precipitated with isopropyl alcohol, washed in ethanol, and resuspended in RNase-free water. RNA quantity and quality were determined by spectrophotometry. Two micrograms of total RNA were used for reverse transcription (RT) and the RT reagent kit method as the instruction book described. cDNA was used for quantitative polymerase chain reaction (PCR) and repeated three times. Quantitative PCR products were synthesized. Each 20 *µ*l SYBR Green reaction system contained 1.0 *µ*l cDNA, forward primer 0.75 *µ*l, and reverse primer 0.75 *µ*l, and the primer concentration is 0.1 *µ*mol/l. The following PCR protocol was applied: 95°C for 60 s, 40 cycles of 95°C for 5 s, and 55°C for 30 s. The results were analyzed by the double Δ*C*_t_ method, which reflects the expression difference based on the cycle threshold of the target gene relative to that of beta-actin (reference gene) in each sample. The specific PCR primers used for all target genes are shown in Table 2.

### 2.8. Staining for SA-β-Galactosidase and the BrdU Analysis

The specific protocol was performed as previously described [3]. X-gal was purchased from Sigma-Aldrich Company (USA), and the BrdU Cell Proliferation Assay Kit was purchased from Chemicon International (Temecula, CA, USA) and used following the manufacturer's protocol.

### 2.9. Statistical Analysis

The statistical analysis was performed using the SPSS 16.0 software package (SPSS Inc., Chicago, IL, USA). Data are expressed as the means ± SEM. To determine significant differences between the COPD patient group and the control group, *t*-tests were used, and to evaluate differences among the three rat groups, one-way ANOVA (analysis of variance) followed by post hoc multiple comparison LSD tests was used. Significance was defined as *p* < 0.05.

## 3. Results

### 3.1. The Expression Levels of P16 and P21 Increased in the Airway Epithelia of COPD Patients

**Figure 1:**
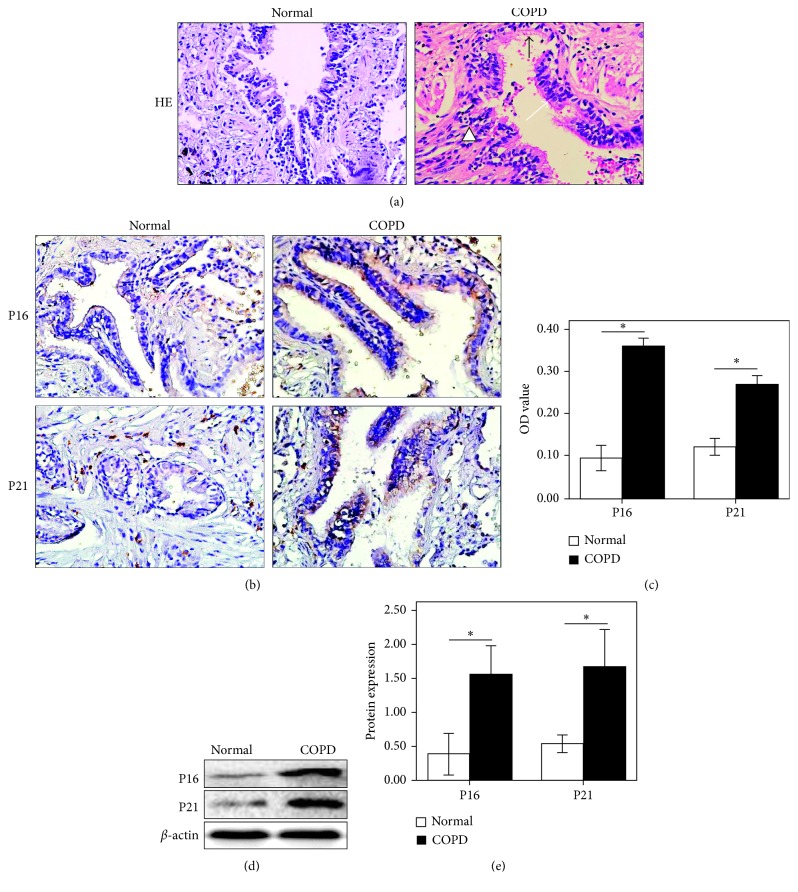
The production of P16 and P21 in the airway epithelium of COPD patients. (a) Micrographs of histological sections of the COPD patients' airway showed the loss of epithelial integrity, apparent deciduous epithelial cells (black arrow), cilia lodging (white arrow), and submucosal and bronchial surrounding fibrous tissue hyperplasia (triangle) by HE staining (HE, 400x). (b) The expression of P16 and P21 in the airway epithelium of patients with COPD and control subjects were detected by IHC (200x). (c) Semiquantitative analysis of P16 and P21 expression in COPD and control groups was conducted by the optical density (OD). (d, e) The expression of P16 and P21 in the airway epithelium of patients with COPD and control subjects were determined by western blotting. ∗*p* < 0.05.

In the lung tissue from COPD patients, pathological changes in the airway epithelium, including an injured epithelium structure, goblet cell hyperplasia, cilia falling off, and thickened basement membranes, were observed (Figure 1). The lung tissue from non-COPD subjects exhibited a normal pulmonary architecture (Figure 1). The P16 and P21 proteins were expressed in both cytoplasm and nuclei of airway epithelial cells by histological analysis (Figure 1), and the expression levels were significantly higher in patients with COPD than those in non-COPD subjects through semiquantitative analysis (Figure 1, *p* < 0.05). It was further found that the protein levels of both P16 and P21 were obviously higher in the COPD patient group than those in the control group (Figures 1 and 1, *p* < 0.05).

### 3.2. CSE Can Induce Human Bronchial Epithelial Cellular Senescence

To detect the effect of the CSE on airway epithelial cells, we treated 16HBE cells with increasing concentrations of the CSE for different durations. In this study, we found that the expression levels of P16 and P21 increased as the CSE concentration increased. In addition, the CSE at a concentration of 2.0% can efficiently increase the protein expression levels of P16 and P21, but there was no significant difference between the 2.0% and 5.0% treatments. Therefore, the optimal CSE concentration was chosen as 2.0% in this study (Supplementary Figures 2A–2C). 16HBE cells were immersed in 2.0% CSE for the indicated time periods (0 h, 3 h, 6 h, and 12 h), and the expression levels of P16 and P21 were detected. The levels of the two proteins reached a maximum at 6 h, and the expression of these markers was prolonged after stimulation for 12 h; accordingly, the best reaction time was 6 h (Supplementary Figures 2D–2F). To further analyze cellular senescence induced by the CSE, SA-*β*-gal was stained in 16HBE cells. It was observed that the percent of SA-*β*-gal-positive cells in the control group (18.25% ± 0.96%) was significantly lower than that in the CSE group (32.25% ± 2.63%) (*p* < 0.05; Supplementary Figures 3A and 3B). In addition, BrdU was used to detect cell proliferation, and the percent of BrdU-positive cells in the control group (30.24% ± 0.89%) was significantly higher than that in the CSE group (19.49% ± 0.62%) (*p* < 0.05; Supplementary Figure 3C). These results suggested that the CSE could promote cellular senescence in bronchial epithelial cells.

### 3.3. O. sinensis Can Inhibit CSE-Induced Cellular Senescence in Bronchial Epithelial Cells

As shown above, the CSE can induce cellular senescence by increasing the production of P16 and P21. To detect the effect of *O. sinensis* on CSE-induced cellular senescence, 16HBE cells were pretreated with *O. sinensis* for 2 h before stimulation with the CSE, and then, P16 and P21 expression levels were examined. The results showed that *O. sinensis* can reduce the increased P16 and P21 expression induced by the CSE at both the protein and mRNA levels, but the levels were still higher than those of the control group (Supplementary Figures 4A–4C). In addition, the percent of SA-*β*-gal-positive cells was 25.25% ± 1.70% in cells pretreated with *O. sinensis*, and there was a significant difference compared with CSE-treated cells (32.25% ± 2.63%), indicating that *O. sinensis* can delay CSE-induced cellular senescence (Supplementary Figures 3A and 3B). The percent of BrdU-positive cells in the cells pretreated with *O. sinensis* (25.48% ± 0.78%) was increased, and there was a statistically significant difference comparison with the CSE group, indicating that *O. sinensis* can improve the proliferation of 16HBE cells (Supplementary Figure 3C). These data suggested that *O. sinensis* could inhibit CSE-induced senescence in bronchial epithelial cells, which are consistent with the results of our previous experiments [5].

### 3.4. O. sinensis Can Inhibit Airway Epithelial Cell Senescence in COPD Rat Models

**Figure 2:**
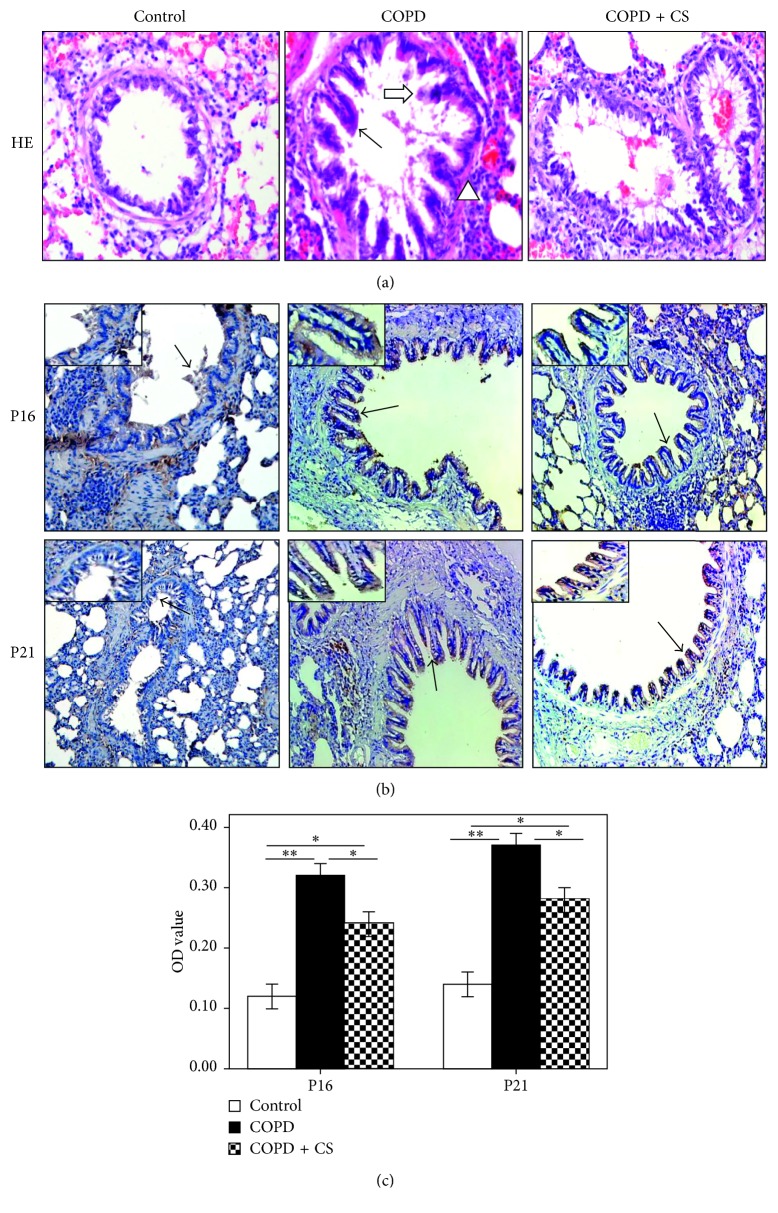
The expression of P16 and P21 in the airway epithelial cells of COPD rat models. (a) The pathological structure changes of airway in COPD rats were observed by HE staining (HE, 200x). In the control group, the airway epithelium structure was complete, and cell fall off, cilia lodging, goblet cell hyperplasia, and phlegm retention were not obvious. In the COPD group, airway epithelial cells fall off partly, and mucosal hyperemia, cilium adhesion or lodging, goblet cell hyperplasia (black arrow), mucus secretion exuberant and large amounts of mucus retention (white arrow), and fibrous tissue hyperplasia (triangle) around the trachea and submucosa were obvious. In the COPD + CS group (*O. sinensis* treatment group), in the airway epithelium, cilia lodging, hyperplasia of goblet cells, mucus gland secretion, and submucosal fibrous tissue hyperplasia were also reduced. (b) The expression of P16 and P21 in the airway epithelium of the COPD group and COPD + CS group were detected by IHC (200x). (c) Quantitative analysis of P16 and P21 expression in the COPD group and COPD + CS group. ∗*p* < 0.05; ∗∗*p* < 0.01.

**Figure 3:**
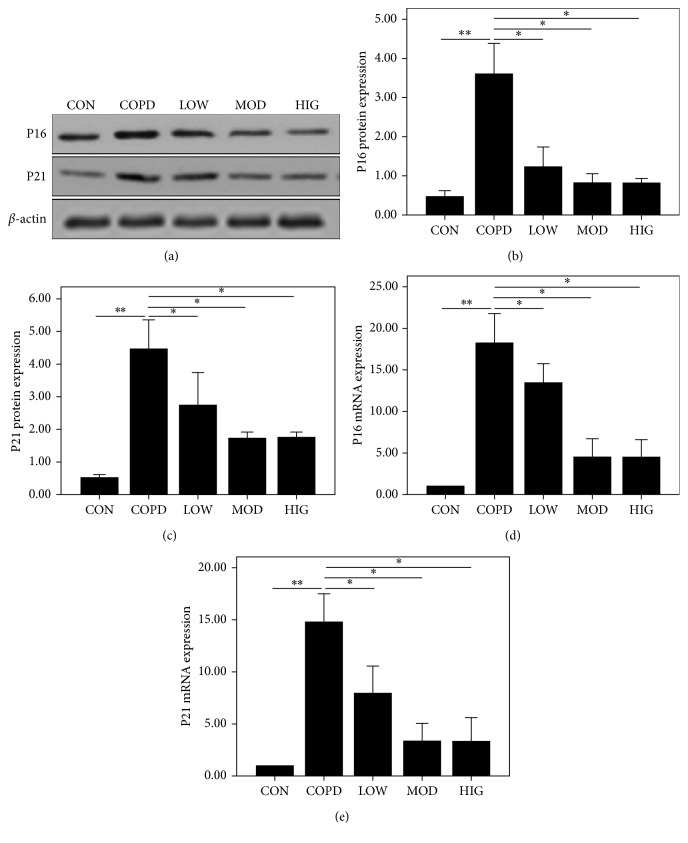
The expression of P16 and P21 in lung tissue of COPD rat models were inhibited by *O. sinensis*. (a) The expression of P16 and P21 in lung tissue of all the groups were detected by western blotting. The quantitative analysis of P16 (b) and P21 (c) in lung tissue of the COPD group and COPD + CS group was conducted. The mRNA expression of P16 (d) and P21 (e) in lung tissue of the COPD group and COPD + CS group was analyzed by qPCR. ∗*p* < 0.05; ∗∗*p* < 0.01.

The lung sections in human COPD patients showed pathological structural changes, including airway epithelial cells that partially fell off, mucosal hyperemia, cilium adhesion or lodging, goblet cell hyperplasia, mucus secretion exuberant and large amounts of mucus retention, and fibrous tissue hyperplasia around the trachea and submucosa. The COPD rat model was successfully constructed based on HE staining of the lung tissue sections (Figure 2). Higher P16 and P21 protein expression levels were observed in both the cytoplasm and nuclei of airway epithelial cells in the COPD model group than in the control group or the *O. sinensis* pretreated group based on IHC staining (Figures 2 and 2). For groups treated with different *O. sinensis* concentrations, the protein and mRNA expression levels of P16 and P21 were remarkably lower in rats treated with a moderate dose and high dose of *O. sinensis* than those in rats treated with a low dose of *O. sinensis* (Figures 3–3). These results indicate that *O. sinensis* could inhibit the expression of P16 and P21 in the airway epithelial cells of COPD rats, indicating that *O. sinensis* may play a protective role in airway epithelial cellular senescence.

## 4. Discussion

Cellular senescence is an important fail-safe mechanism that mainly occurs in response to stress and telomere erosion [14], and senescence can be observed in tumors and loss-of-function disorders, such as Alzheimer's disease, atherosclerosis, osteoporosis, asthma, and COPD. P16 and P21 are involved in cell senescence by inhibiting cyclin-dependent kinases and thereby negatively regulate cell proliferation [9, 15]. As a deeper understanding of COPD pathogenesis is obtained, more and more scholars recognize that cellular senescence not only is present in the COPD lungs but also can explain several key features of the disease. There is a growing realization that COPD involves several processes observed in aging and cellular senescence [8, 16]. In this study, we found increased levels of P16 and P21 expression in the airway epithelial cells of COPD patients; our finding was in accordance with those of our previous study [5]. These proteins have multiple roles in the pathogenesis of the main COPD manifestations, particularly in the propagation of a proinflammatory phenotype, the loss of reparative potential, and the amplification of oxidative stress, which all ultimately lead to tissue damages [17, 18]. A pathophysiological role for cellular senescence in COPD has not been conclusively shown.

Although the mechanism of cellular senescence involved in COPD is not clear in clinical settings, a natural drug could be a good choice for treatment. *O. sinensis* is a fungus endemic to Himalayan alpine habitats at an elevation of 3600–5000 m [19]. *O. sinensis* preserves the intestinal mucosal barrier and may be an adjunct therapy in an endotoxin-induced sepsis rat model based on a pilot study [20, 21]. In this study, we found that *O. sinensis* could indeed inhibit CSE-induced cellular senescence in vitro and in COPD rat models by reducing the expression of P16 and P21. Unfortunately, its mechanism of action is not clear. Perhaps, the most important pathogenic contribution of cellular senescence to the disease process is the enhancement of the inflammatory phenotype by cytokines released from senescent cells, which may underlie self-propagating processes in the COPD lung. A clear implication is that senescent cells, notably progenitor cells, have decreased regenerative properties [22], potentially limiting the ability of the lungs to recover after decades of injury caused by cigarette smoke. Our previous study demonstrated that the CSE could induce cellular senescence in human bronchial epithelial cells, and the ROS/PI3K/AKT/mTOR signaling pathway may play an important role in this process [5]. In a cellular model of hypoxia, cells treated with *O. sinensis* (250 *μ*g/ml) significantly attenuated oxidative stress in hypoxia [19]. This attenuation was attributed to Nrf2-ARE pathway activation, reduced NFκB activity, and increased oxygen availability via HIF1 signaling mechanisms. Furthermore, higher levels of antioxidants and anti-inflammatory mediators, for example, TGF-beta, HO1, and MT, in *O. sinensis*-treated cells might also be responsible for the tolerance of cells to hypoxia [19]. An additional study has shown that *O. sinensis* extracts are highly cytotoxic to human colorectal carcinoma RKO cells and inhibit the growth of tumors in a xenograft model. The antitumor effect of *O. sinensis* has been associated with the induction of cell cycle arrest and mitochondrial-mediated apoptosis [23]. Our research suggests that the CSE could induce cell senescence in vitro and in COPD rats. Therefore, we speculate that *O. sinensis* may delay cellular senescence via several biological processes by inhibiting P16 and P21 protein expression.

In conclusion, the CSE can induce cellular senescence, and *O. sinensis* can alleviate CSE-induced cellular senescence. This study indicates that *O. sinensis* could be the basis for new therapeutic strategies in COPD. However, the specific mechanism underlying the effects of *O. sinensis* needs further research.

## Figures and Tables

**Table 1:** Clinical characteristics of normal subjects and COPD patients.

	Normal subjects	COPD patients
Male/female	14/16	15/15
Age (years)	58.65 ± 4.72	60.54 ± 5.68
FEV1 (% predicted)	88.21 ± 10.54	56.42 ± 17.43^∗^
FVC (% predicted)	85.73 ± 7.54	78.68 ± 17.92
FEV1/FVC (%)	85.43 ± 11.62	50.86 ± 16.43^∗^

Values are presented as means ± SEM. ^∗^ *p* < 0.05 versus controls. FEV1, forced expiratory volume in one second; FVC, forced vital capacity; SEM, standard error of the mean.

**Table 2:** The sequences of PCR primers used in this study.

Primer	Forward (5′–3′)	Reverse (5′–3′)
P16		
Rat	CTACTCTCCTCCGCTGGGAA	GGCCTAACTTAGCGCTGCTT
Human	CTTGGTGACCCTCCGGATTC	CCACGAGATGTGAACCACGA
P21		
Rat	CAGGCTCAGGAGTTAGCAAGG	TCAACACCCTGTCTTGTCTTCG
Human	AGTACCCTCTCAGCTCCAGG	TGTCTGACTCCTTGTTCCGC
*β*-actin		
Rat	ATGATTCATCCCACGGCAAG	CTGGAAGATGGTGATGGGTT
Human	CCGTTGCCCTGAGGCTCTTT	CCTTCTGCATCCTGTCAGCAA

## Supplementary Materials

Supplementary Materials There are two aspects in this part. One is the explanation for the choice of time points the 16HBE cells are exposed to the CSE, and the other is several diagrams for the cell experiment.
