# Supplementary material for: The Function of Ophiocordyceps sinensis in Airway Epithelial Cell Senescence in a Rat COPD Model
Source: Can Respir J. 2018 Apr 1;2018:6080348. doi: 10.1155/2018/6080348 (PMC5902013; doi:10.1155/2018/6080348)

**Figure 1 the explanation to the choice of time point in vitro experiment.**

In our previous experiment, we have found there was no statistic difference on both the P16 and P21 protein expression after exposure to CSE for 6, 12 48 and 96 hours. Therefore the time point was 0, 3, 6 and 12 hours at last.

**Figure 2 The expression of P16 and P21 in airway epithelial cells treated with CSE.** (**A**) The airway epithelial cells were stimulated with CSE at different concentrations, and then the productions of P16 as well as P21 were examined by Western blotting. The expression of P16 (**B**) and P21(**C**) was stable at the concentration of 2%. (**D**) 16HBE cells were treated with 2% CSE for different time, and the expression of P16 and P21 were detected by Western blotting. The optimal time for the expression of P16 (**E**) and P21 (**F**) was 6 hours.

**Figure 3 The effect of O. sinensis on cell senescence and proliferation of 16HBE cells.** (**A**) The cells were fixed and stained with SA-β-gal, and (**B**) positive SA-β-gal cells in CSE group and CSE+CS group（O. sinensis treatment group） were quantified. (**C**) The proliferative phase S of cells were detected by BrdU staining. The cells were fixed before stained with BrdU, and the BrdU positive cells were quantified. *p<0.05.

**Figure 4 The effect of O. sinensis on P16 and P21 expression in 16HBE cells.** (**A**) The 16HBE cells were pre-treated with O. sinensis (100 μg/ml) for 2h before stimulated with 2% CSE for 6h, and the expressions of P16 and P21 were detected by Western blotting. (**B**)The quantitative analysis of P16 and P21 in cells treated with CSE and/or O. sinensis were conducted. (**C**) The mRNA expression of P16 and P21 in cells treated with CSE and/or O. sinensis were analyzed by qPCR. *p<0.05; **p<0.01.

Figure 1


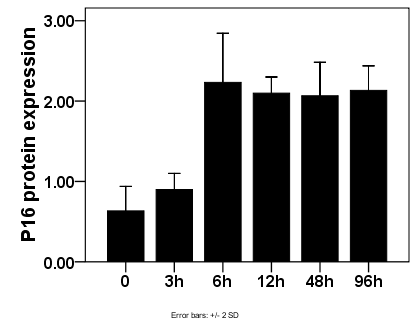


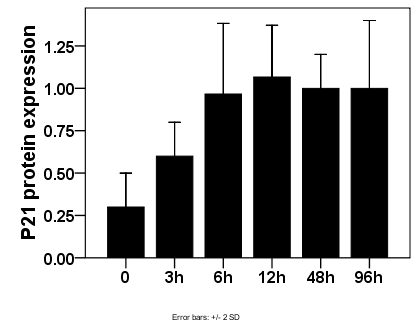


Figure 2


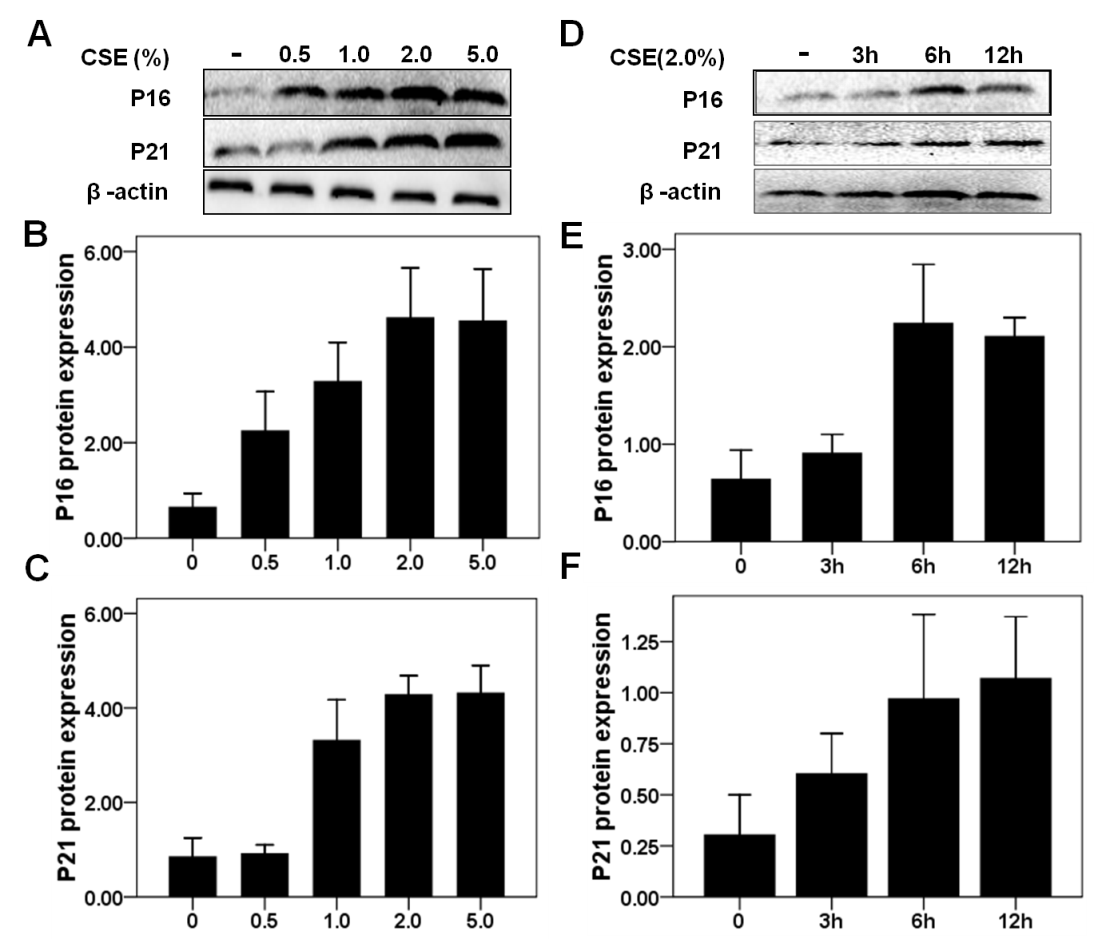


Figure 3


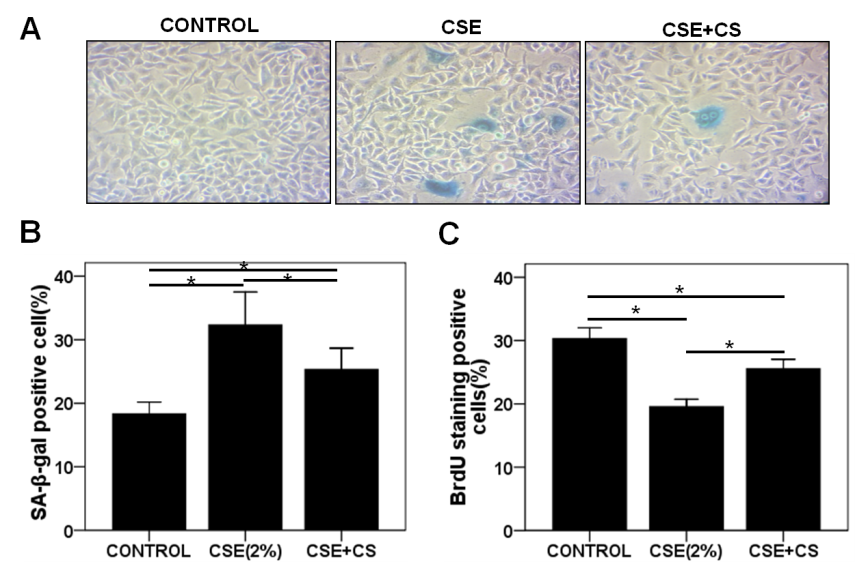


Figure 4


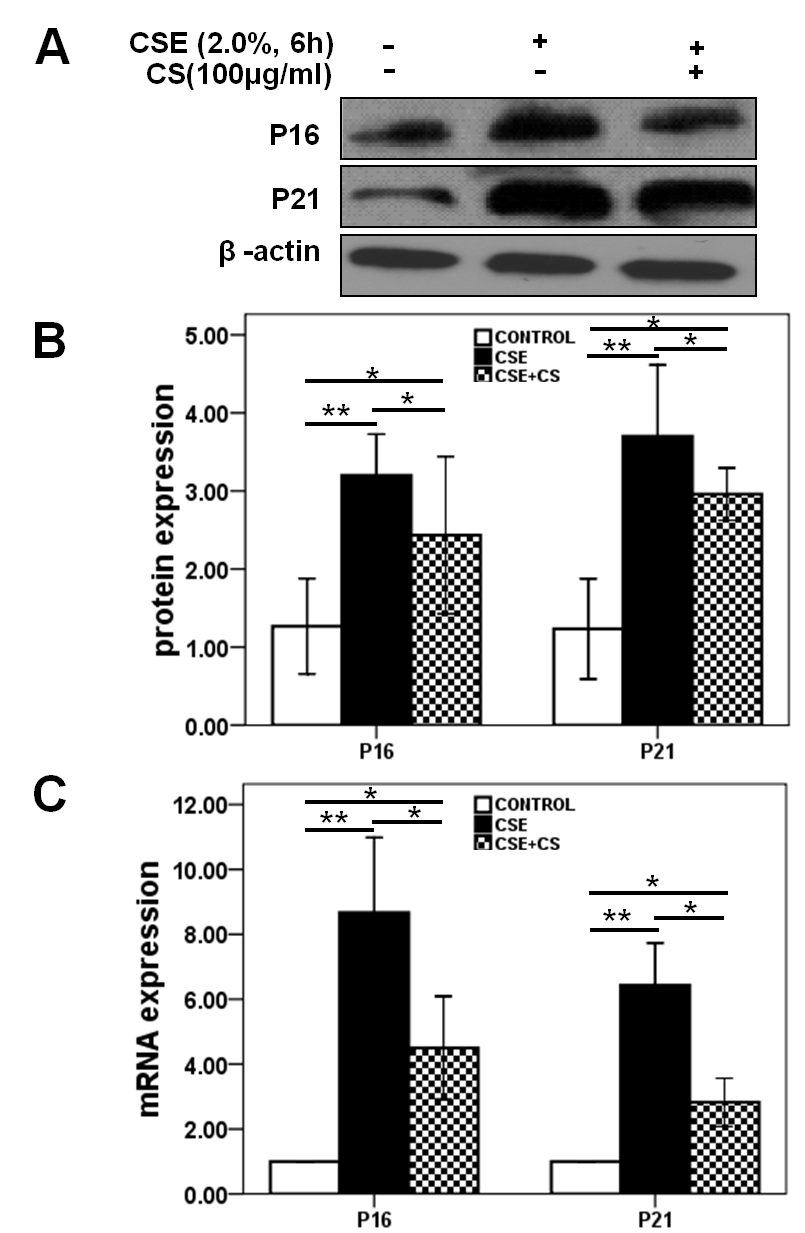

Supplement: Supplementary Materials — There are two aspects in this part. One is the explanation for the choice of time points the 16HBE cells are exposed to the CSE, and the other is several diagrams for the cell experiment. [file 6080348.f1.docx]
